# Surface Modification of Porous Polyethylene Implants with an Albumin-Based Nanocarrier-Release System

**DOI:** 10.3390/biomedicines9101485

**Published:** 2021-10-16

**Authors:** Jonas Eckrich, Niklas Hoormann, Erik Kersten, Keti Piradashvili, Frederik R. Wurm, Martin Heller, Sven Becker, Toni Anusic, Juergen Brieger, Sebastian Strieth

**Affiliations:** 1 Department of Otorhinolaryngology, University Medical Center Mainz, Langenbeckstrasse 1, 55131 Mainz, Germany; Niklas.Hoormann@unimedizin-mainz.de (N.H.); Martin.Heller@uni-mainz.de (M.H.); Brieger@uni-mainz.de (J.B.); Sebastian.Strieth@UKBonn.de (S.S.); 2 Department of Otorhinolaryngology, University Medical Center Bonn (UKB), Venusberg-Campus 1, 53127 Bonn, Germany; 3 Max Planck Institute for Polymer Research (MPIP), Ackermannweg 10, 55128 Mainz, Germany; Erik.Kersten@gmx.net (E.K.); Piradashvili.k@gmail.com (K.P.); Wurm@mpip-mainz.mpg.de (F.R.W.); 4 Sustainable Polymer Chemistry, Department of Molecules and Materials, MESA+ Institute for Nanotechnology, Faculty of Science and Technology, Universiteit Twente, P.O. Box 217, 7500 AE Enschede, The Netherlands; 5 Department of Otorhinolaryngology, Head and Neck Surgery, University of Tübingen, Elfriede-Aulhorn-Str. 5, 72076 Tübingen, Germany; Sven.Becker@med.uni-tuebingen.de; 6 Institute of Medical Biostatistics, Epidemiology and Informatics (IMBEI), University Medical Center, Obere Zahlbacher Str. 69, 55131 Mainz, Germany; Toni.Anusic@imbei-mainz.de

**Keywords:** porous polyethylene, biomaterial, material science, albumin nanocarriers, tissue engineering, release kinetics, dorsal skinfold chamber, fluorescence microscopy

## Abstract

Background: Porous polyethylene (PPE) implants are used for the reconstruction of tissue defects but have a risk of rejection in case of insufficient ingrowth into the host tissue. Various growth factors can promote implant ingrowth, yet a long-term gradient is a prerequisite for the mediation of these effects. As modification of the implant surface with nanocarriers may facilitate a long-term gradient by sustained factor release, implants modified with crosslinked albumin nanocarriers were evaluated in vivo. Methods: Nanocarriers from murine serum albumin (MSA) were prepared by an inverse miniemulsion technique encapsulating either a low- or high-molar mass fluorescent cargo. PPE implants were subsequently coated with these nanocarriers. In control cohorts, the implant was coated with the homologue non-encapsulated cargo substance by dip coating. Implants were consequently analyzed in vivo using repetitive fluorescence microscopy utilizing the dorsal skinfold chamber in mice for ten days post implantation. Results: Implant-modification with MSA nanocarriers significantly prolonged the presence of the encapsulated small molecules while macromolecules were detectable during the investigated timeframe regardless of the form of application. Conclusions: Surface modification of PPE implants with MSA nanocarriers results in the alternation of release kinetics especially when small molecular substances are used and therefore allows a prolonged factor release for the promotion of implant integration.

## 1. Introduction

Craniomaxillofacial defects may occur due to invasive tumors, as well as a result of extensive trauma, infection, or through congenital deformities. These defects can lead to an intensely disfigured appearance resulting in social exclusion, isolation, and loss of self-esteem for the individual. Apart from epithetics, a variety of plastic surgical techniques are available to facilitate a normal physical appearance by plastic reconstruction. Allografts like rib cartilage [1,2] or bone tissue derived from the iliac crest [3,4] can be used to facilitate the framework of three-dimensional structures. However, in some cases, defects due to their extent may require the use of foreign material.

Porous polyethylene (PPE) implants have often been successfully used for the reconstruction of bone and cartilaginous tissue of the craniomaxillofacial region [5,6] and have proven to be suited as a surgical tool for auricular [7] as well as nasal [8,9,10] or maxillofacial reconstruction [11]. PPE is easy to harvest and provides an adequate amount of durability and strength to serve as a structural base for reshaping three-dimensional tissue formations. However, depending on the localization of implantation a significant percentage of insufficient integration into the host tissue results in short-term or long-term graft failure [12,13]. Tissue engineering strategies like increase of porosity and pore interconnectivity [14,15] as well as combining PPE with biodegradable polyesters and/or cellular precolonization has shown to improve tissue formation within the implant [16]. Angiogenesis into the polyethylene implant has shown to be a prerequisite for a successful integration of the scaffold into the surrounding tissue as vessel ingrowth enables cellular colonization resulting in integration of the implant [17,18]. As oxygen transport from blood vessels of the surrounding tissue by diffusion is limited to a distance of approximately 150–200 μm [19] survival of cells within the implant is depending heavily on blood vessel ingrowth.

Various pro-angiogenic and immune-modulating factors have shown the potential to promote the ingrowth of blood vessels into the implant, thus enabling a dense cellular colonization and/or lower inflammatory response and to promote scaffold integration [14,20,21,22,23]. However, for most factors, a sustained release and constant tissue level are prerequisites for promotion of implant integration [24,25,26].

A long-term gradient can be realized by a sustained release. Drug delivery systems (DDS) are used to achieve this effect [27]. The goal of a DDS is the delivery of a biologically active substance to the target site in an adequate dose over a desired period of time [28,29,30,31]. Contrary to these effects, a burst release of a substance discharges a large amount of the specific drug with only short-term biological effects [31]. Since growth factors usually diffuse rapidly from the actual target site or are quickly degraded or deactivated, a DDS has the goal to protect them from degradation without impairing their bioactivity [28,32,33]. For some factors, surface coating [34] as well as release from hydrogels [20,23,35,36] have shown their potential as DDS by realizing a long-term gradient. The protection of sensitive biomolecules by encapsulation in microspherical or nanospherical capsules synthesized from exogenous substances has shown to be an alternative method to prevent the burst release [33].

Albumin has proven to be a promising DDS for various medical applications without an extensive risk of immunogenicity or toxicity. Albumin is the protein with the highest concentration in plasma. Its half-life in the bloodstream is approximately 19 days, it has a stable molecular structure at a pH between 4 and 9 and can tolerate temperatures of 60 °C degrees for up to 10 h. Under physiological conditions albumin is degraded by intracellular uptake and decomposition in lysosomes. From a biochemical standpoint albumin has many binding possibilities in the form of reactive amino, carboxyl, and thiol groups as well as a high proportion of charged amino acids [37,38,39].

Taking these properties into consideration, albumin is used as DDS by covalent and non-covalent binding of substances, as a shell for micro- and nanocarriers, as fusion protein, and for targeting [37,38,40,41,42,43]. Moreover, nanoparticulate albumin DDS have been established in the form of nanoparticle albumin bound (nab)-paclitaxel, which uses nanoparticles as a carrier for paclitaxel and can thus achieve higher accumulation in tumor tissue as well as reduced systemic toxicity [44,45,46]. Thus, the use of albumin for surface modification in the field of tissue engineering seems like a promising approach. Preliminary work by Piradashvili et al. showed that albumin nanocarriers, crosslinked by orthogonal photoclick tetrazole–ene chemistry with a high loading efficiency of (91%), an average size of approximately 300 nm and a zeta potential of about −30 mV, showed no cytotoxicity, no aggregation in blood plasma, and no leakage of the encapsulated substance in vitro. Furthermore, the albumin-nanocarriers were absorbed by dendritic cells and could be enzymatically degraded by trypsin [47].

Thus, the modification implants using a biocompatible, protein-based nanocarrier, capable of realizing controlled factor release, which can be bound onto the PPE implants was conducted.

The goal of the present study was the in vivo characterization of coated PPE implants, surface-modified with nanocarriers based on murine serum albumin (MSA). Modified implants were subsequently implanted into the skinfold of mice. To investigate the ability of the albumin nanocarriers to achieve a long-term gradient both encapsulated low molar mass (Sulforhodamine 101 (SR101) [606.71 Da]) and high molar mass (Sulforhodamine-linked Etanercept [~150 kDa]) molecules were encapsulated into the protein nanocarriers upon implant modification. Thus, in vivo, the release kinetics of both small molecular substances as well as macromolecules were studied. Analysis was performed using intravital-fluorescence microscopy for a total of ten days after PPE implantation. In control cohorts, the implant surface was coated with the homologue non-encapsulated cargo-substance.

## 2. Materials and Methods

### 2.1. Porous Polyethylene Implants

PPE implants (MEDPOR, Stryker Craniomaxillofacial, Portage, MI, USA) with a pore size of 100–200 µm were cut to a scaffold size of 50 mm × 3 mm × 0.1 mm under sterile conditions and again sterilized upon implantation by steam sterilization (5050 ELVD, Tuttnauer, Breda, Netherlands) upon further processing. After steam sterilization the implants showed no altered structure and sample implants were negatively tested for endotoxin contamination (QCL-1000TM Endpoint Chromogenic LAL Assay, Lonza Group, Basel, Switzerland).

### 2.2. Nanocarrier Preparation and Implant Modification

Nanocarriers from murine serum albumin (MSA) were prepared using an inverse miniemulsion technique as previously described [47] with minor modifications (MSA was used instead of bovine serum albumin and modified with 4-(2-phenyl-2H-tetrazol-5-yl (TET) benzoic acid).

### 2.3. Modification of MSA

To start the coupling reaction of 4-(2-phenyl-2H-tetrazol-5-yl)benzoic acid (TET) with mouse serum albumin (MSA) with a degree of functionalization of 10% according to 51 lysine groups (HSA: 60 lysine groups), 50 mg of the MSA were dissolved in 3 mL dimethylsulfoxide (DMSO). Stock solutions of TET, 1-ethyl-3-(3-dimethylaminopropyl)carbodiimide (EDC) and 4-(dimethylamino)pyridine (DMAP) were prepared in DMSO, because of very small amounts. TET and DMAP were added to the stirring mixture. After achieving a homogenous solution, EDC was added. The reaction mixture was stirred for 48 h. To remove the side products, the reaction mixture was diluted with 10 mL demineralized water and dialyzed against demineralized water for 4 days (MWCO 3500 Da). After lyophilization, 45 mg of the modified MSA was obtained and the determination of the amount of TET in the protein was done by UV/Vis-spectroscopy. The amounts used for the esterification reaction are shown in Table 1.

### 2.4. Nanocarrier Preparation

The formation of the protein nanocarriers was performed by using an inverse mini-emulsion (water in oil). For the water phase: 12.5 mg of the TET-functionalized protein was dissolved in 250 µL phosphate buffer Na2HPO4/KH2HPO4 (pH = 7.6). If a dye was used: reduce phosphate buffer volume to 225 µL and add 25 µL of dye solution in demineralized water. For the oil phase: 17.9 mg of surfactant P((E/B)-b-EO) was dissolved in 3.75 g of cyclohexane and the mixture was stirred until dissolved. The oil phase was added drop-wise to the stirred water phase. To form the inverse mini-emulsion, ultrasound was used: the mixture was cooled with an ice bath, ultrasonified for 3 min at 70% amplitude with pulse cycles of 20 s sonication and 10 s pauses. A second oil phase solution was prepared with 1.0 mg of surfactant dissolved in 0.5 g cyclohexane and the di-norbornene crosslinker (100-fold molar excess to the TET-protein). After dropwise addition of the second oil phase to the stirred mini-emulsion, the reaction mixture was transferred to a quartz glass tube. The peristaltic pump had a flow rate of 2.5 mL/min and the reaction mixture was irradiated with 254 nm for 30 min using commercially available TLC-UV-lamp. The synthesized nanocarriers were purified by repetitive centrifugation: 3 times with 4000 rpm for 20 min and removal of the supernatant (excess surfactant and cross-linker) and re-dispersion in pure cyclohexane. To transfer the nanocarriers into the aqueous phase, 6 times 100 µL of the cyclohexane dispersion was added via a micro pipette to 5 g of a 0.1 wt.% aqueous SDS solution in an ultrasound bath. After that the samples were stirred in open vials for 24 h at room temperature to evaporate the cyclohexane. The purification was done by centrifugation: 3 times with 4000 rpm for 20 min and removal of the supernatant (excess surfactant) and re-dispersion in pure demineralized water.

**Figure 1:**
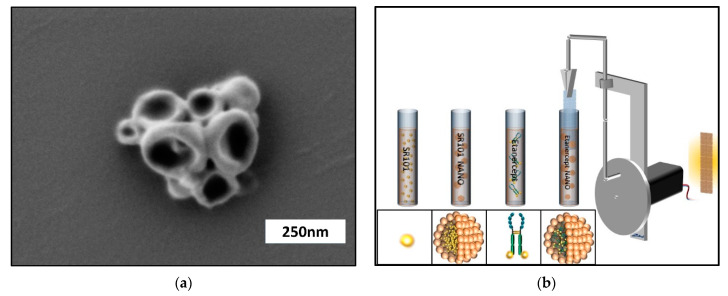
(**a**) Scanning electron microscopy image of albumin nanocarriers after synthesis. (**b**) Dip coating of the implants. PPE implants were coated with SR101, SR101 enclosed by nanocarriers, native SR101-labeled Etanercept or SR101-labeled Etanercept enclosed by nanocarriers respectively.

The obtained nanocarriers were characterized by dynamic light scattering (DLS) with average diameters of ca. 197 nm in cyclohexane and by scanning electron microscopy (SEM). (Figure 1a)

### 2.5. Coating of PPE Implants

The modification of the implant is physically by the application of a dip-coater and a cyclohexane miniemulsion. Since polyethylene is highly hydrophobic, coating of the PPE implants was achieved by dip-coating of the dispersion of the protein nanocarriers in cyclohexane. Due to the presence of the hydrophobic groups in the carrier and the used surfactant, we achieved a sufficient adhesion to the hydrophobic PE-substrate. A dip-coater was used for the deposition of the protein nanocarriers on the surface of the porous PE by dipping into a 0.75 wt.% dispersion in cyclohexane for 10 times and turning the sample around and dipping another 10 times to obtain a homogenously distributed coating. Before further processing the even distribution of implant coating was evaluated using fluorescence microscopy (Olympus BXFM, OLYMPUS DEUTSCHLAND GmbH, Hamburg, Germany). If certain areas did not display an even distribution of fluorescent dye, they were excluded from further experimentation. Evenly coated implants were subsequently cut to a scaffold size of 3 mm × 3 mm × 0.1 mm under sterile conditions. Randomly selected samples of PPE with each specific modification (*n* = 3) were re-evaluated regarding endotoxin contamination as mentioned above. The prepared PPE implants were divided into four groups and coated with nanocarriers bearing SR101, as well as nanocarriers bearing SR101-labeled Etanercept (ENBREL, Pfizer Pharma GmbH, Berlin Germany). To allow the quantification of the effects of encapsulation on the pharmacokinetics the homologue non-encapsulated cargo-substance (SR101; SR101-labeled Etanercept) was bound to PPE implants by dip coating (Figure 1b).

### 2.6. Animals

For the PPE implantation, male C57BL/6J mice (Janvier, Le Genest-Saint-Isle, France), aged > 6 weeks (>25 g body weight) were used as experimental animals. Chamber preparation was performed as recently described by our working group [48,49]. After chamber preparation, in order to avoid injuries inflicted by other mice, animals were isolated. During the course of experimentation animals had free access to tap water as well as standardized food (ssniff, Spezialdiaeten GmbH, Soest, Germany). All experimental procedures were performed according to institutional and governmental guidelines and animal experiments were approved by the Landesuntersuchungsamt Rheinland Pfalz (G 15-1-066). Furthermore, all people involved in the experimental course were qualified to perform experimental procedures on laboratory animals.

Animals were divided into four cohorts and received specific PPE implants coated with either nanocarriers bearing SR101, native SR101, as well as nanocarriers bearing SR101-linked Etanercept, or native SR101-linked Etanercept respectively.

### 2.7. Dorsal Skinfold Chamber Preparation

For the surgical procedure, animals were anesthetized by intraperitoneal injection of 0.1 mg/g ketamine (Ketanest, Pfizer Pharma GmbH, Berlin, Germany) and 0.01 mg/g xylazine (Rompun; Bayer, Leverkusen, Germany). After chamber implantation, weight and health conditions of every animal were monitored on a daily basis. A weight loss > 20% of the postoperative bodyweight, signs of inflammation, or behavioral changes indicating pain or sickness were determined as dropout criteria.

**Figure 2:**
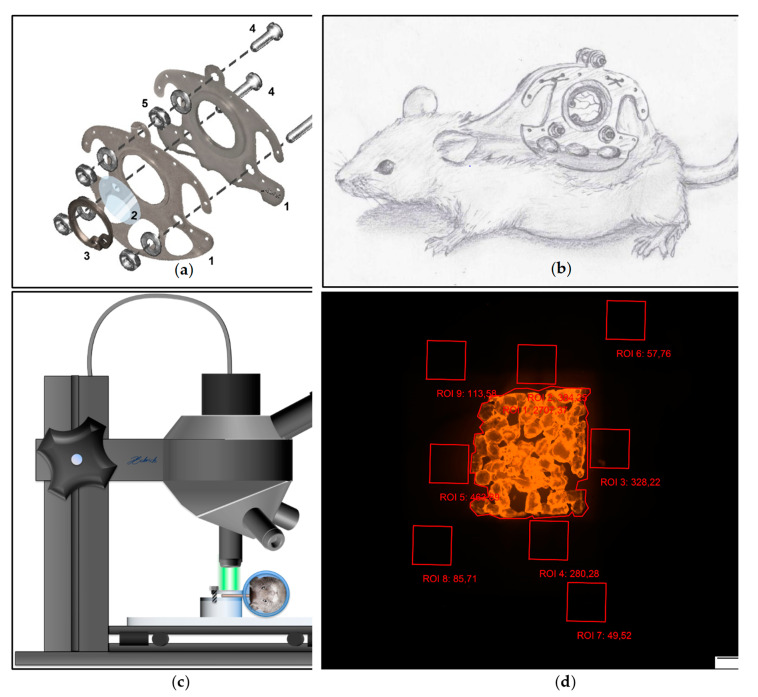
(**a**) Dorsal skinfold chamber consisting of the two titanium chambers (1), the cover glass (2), the retaining ring (3), and the sweeping screws (4) with associated nuts (5). (**b**) Schematic drawing of a mouse after chamber preparation. (**c**) In vivo microscopy setup. (**d**) Positioning of ROI for analysis of fluorescence intensity during fluorescence microscopy.

Before the surgery began, total anesthesia was substantiated by the loss of positional as well as corneal and interdigital reflexes. After sufficient depth of anesthesia was achieved, the surgical procedure was carried out as previously described [44]. After mechanical and chemical depilation of the dorsal skin, the two opposing sides of the chambers’ titanium frame (Figure 2a) were sutured onto a dorsal skin double layer of the anesthetized animal (Figure 2b). After incision of the skin tissue at the predetermined positions, both chamber frames were screwed together with sweeping screws. In the area of the chamber window the skin as well as the underlying musculocutaneus tissue were surgically removed using micro scissors and a micro forceps. A cover glass (Hecht Assistent, Glaswarenfabrik Karl Hecht GmbH & Co KG, Sondheim, Germany) was placed on the musculocutaneous tissue of the opposing layer within the chamber window and was subsequently fixed with a flexible retaining ring. Animals subsequently received a single-shot dose of subcutaneously applied 3 μg Flunixin-Meglumin (Finadyne RPS 83 mg/mL, MSD Animal Health Innovation GmbH, Schwabenheim, Germany) per gram bodyweight. Additionally, animals received subcutaneously injected Marbofloxacin (Marbocyl 2%, Vetoquinol GmbH, Ismaning, Germany) (2 μg/g bodyweight) for five days. After recovery from the surgery for 48 h, the PPE scaffolds were implanted into the musculocutaneous tissue within the chamber window by removal of the cover glass. For postoperative analgesia, the animals received 0.1 mg/mL Tramadol (Tramadol-ratiopharm, ratiopharm GmbH, Ulm, Germany) with the daily-applied drinking water.

### 2.8. In Vivo Microscopy

For the in vivo fluorescence microscopy, the animals were immobilized in an acrylic glass tube. The chamber was positioned horizontally underneath the in vivo microscope (Olympus BXFM, OLYMPUS DEUTSCHLAND GmbH, Hamburg, Germany) and fixed with screws onto the acrylic platform (Figure 2c). After immobilization of the chamber, a multi-image-array (MIA) covering the entire chamber field was taken using a Cy3 filter (Excitation [Ex]: 545 nm; Emission [Em] 605 nm). The MIAs were analyzed using CellSens Dimension (OLYMPUS DEUTSCHLAND GmbH, Hamburg, Germany) [50]. Nine regions of interest (ROI) measuring 1 mm^2^ were defined. One ROI was positioned covering the implant surface (ROI 1), four above the tissue surrounding the implant (ROI 2–5) as well the tissue distancing the implant at least 200 µm (ROI 5–9). Fluorescence intensity (in arbitrary units [AU]) was repeatedly analyzed and mean of fluorescence intensity was measured for the ROI covering the implant surface as well as the ROI neighboring the implant and the ROI distancing at least 200 µm from the implant surface (Figure 2d). The range of detectable fluorescence intensity ranged from 0 AU to a maximum of 4096 AU.

For simplification of identification, the experimental cohorts were named “SR native” (PPE covered with non-encapsulated, “native” SR101) and “SR nano” (PPE covered with SR101 encapsulated in Albumin nanocarriers) as well as “Etanercept native” (PPE covered with non-encapsulated, “native” SR101-labeled Etanercept) and “Etanercept nano” (PPE covered with SR101-labeled Etanercept encapsulated in Albumin nanocarriers) throughout the manuscript.

### 2.9. Analysis of the Vascular Network

To evaluate the vascular network after implantation, MIA were reduced in brightness and overall contrast using cellSens Dimension (OLYMPUS DEUTSCHLAND GmbH, Hamburg, Germany) till detection of the vascular network was possible. Images of day 1, 5, 8, and 10 after implantation were then reevaluated regarding vascular density, if the vascular network was visible and image proved to be sufficient for further analysis (Figure S1a). The vascular network within the ROI directly adjacent to the implant (ROI 2–5) was then traced by hand using the “Polyline” function in cellSens Dimension thus measuring the vascular density (vessel length/mm^2^) in every ROI (Figure S1b). Images were subsequently exported as jpg. after removal of the ROI frame (Figure S1c). After cropping of the image, the blue channel was isolated using IrfanView (Figure S1d) [51]. The images were imported into the Java-based open-source software Fiji [52]. Image were converted into black and white using Fiji’s “Make binary” function and subsequently Skeletonized using the “Skeletonize” function (Figure S1e). The Skeleton was then analyzed using the “Anlalyze Skeleton” function and values of branches and junctions (Figure S1f) of every image were calculated and exported into Excel files.

### 2.10. Data Management and Statistical Analysis

To answer the two general questions: (a) Does the mean fluorescence per ROI differ significantly between two mouse cohorts? and (b) Does the mean decrease of the fluorescence per ROI differ significantly between two mouse study groups?—a linear mixed model was used. The linear mixed model was implemented utilizing R (version 3.4.2) with the lmerTest package (version 3.1.0.).

The question (a) and (b) were respectively answered by including the mouse cohort (a) and the interaction between mouse cohort and time (b) as fixed effects into the mixed model; furthermore, the time itself was also incorporated as a fixed effect to adjust for a time-dependent association. As repeated measures over time of the same subject are often heavily correlated—in this case the fluorescence of each mouse over 10 days—this correlation must be considered in the statistical analysis. For that a random intercept was included into the mixed model—in addition to the fixed effects—to adjust for the subject-dependent correlation. Furthermore, for each random intercept a corresponding random slope was fitted to account for the time-dependent correlation per subject. Applying this weighting approach at our mixed model, the random intercept and slope correspond to the mouse and time, respectively.

Notably for the sake of convenience and due to similarity, the ROI 2, 3, 4, 5 and 6, 7, 8, 9 were respectively averaged, resulting in the three different regions of interest: ROI 1, ROI 2–5, and ROI 6–9. Instead of the absolute fluorescence per mouse, the relative fluorescence (0–100%) was analyzed. For each mouse, its relative fluorescence was calculated by dividing the day-dependent absolute fluorescence by the absolute fluorescence at day 1. This resulted in a scaled fluorescence of 100% at day 1 for each mouse granting a more consistent interpretation between the mice and the different cohorts.

Furthermore, compared to the absolute fluorescence, the relative fluorescence ensures much better normal distributed residuals of the linear mixed model, a better linearity between the fluorescence and fixed effects and a more intuitive interpretability of the estimates. Note that in the cohort “SR native” the fluorescence drops exponentially from day 1 to day 2 and only then follows a linear decrease, violating the previously described linearity. Therefore, the linear mixed model for all cohorts was divided into the two-time intervals “day 1–2” and “day 2–10”.

Since effects of the nanocarriers were primarily to be expected in the implant (ROI 1) and secondarily in other parts of the chamber (ROI 2–5 and ROI 6–9), the confirmatory part in this analysis covers the following questions:

Is there a significant difference regarding the decrease of the fluorescence over 10 days in ROI 1 between the cohorts “SR native” and “SR nano”?

Is there a significant difference regarding the decrease of the fluorescence over the first hour in ROI 1 between the cohorts “SR native” and “SR nano”?

Is there a significant difference regarding the decrease of the fluorescence over 10 days in ROI 1 between the cohorts “Etanercept native” and “Etanercept nano”?

Each question was described by the null hypothesis H0: βinteraction = 0 vs. the alternative hypothesis HA: βinteraction ≠ 0 where βinteraction denotes the difference of regression slopes between the two corresponding cohorts. The first two questions were answered for the time intervals day 1–2 and day 2–10 resulting in 2 × 2 = 4 hypotheses. The 3rd question generates only one null hypothesis as the mixed model can be run for the whole-time interval. Here the time interval corresponds to 0–60 min divided into 15 min measurement time points.

As five null hypotheses were to be tested for statistical significance, the global significance level of 5% was divided into five local significance levels of 1% using the Bonferroni correction. Hence, each null hypothesis is rejected if its corresponding *p*-value is ≤0.01 declaring the difference of regression slopes “significant”.

The explorative part in this analysis deals with the same confirmatory questions but for different ROI. As in this case no adjustments for multiple testing are done, *p*-values will be simply given.

Comparative analysis of vessel density as well as the number of vessel junctions and vessel branches was performed using GraphPad Prism™ (GraphPad Software, Inc., La Jolla, CA, USA). Absolute values as well as relative changes (%) where compared using the Mann Whitney *u* Test.

## 3. Results

### 3.1. Study Cohort

62 animals were included in the investigational protocol. The total dropout rate was 28.5% of which 33.4% dropped out due to a weight loss exceeding 20% of the postoperative bodyweight and 66.6% due signs of inflammation or behavioral changes indicating pain or sickness. In 81.0% of the mice included, the obtained images were of sufficient quality for off line analysis while 12.7% were excluded due to a partial drying out of the chamber and 3.2% due to either non-adherence or hemorrhage within the chamber window. In the “SR nano” group *n* = 9 individuals were evaluated while in all other study groups the cohort comprised of *n* = 8 individuals.

Study group specific dropout rates are rather similar though statistical comparison is not sufficiently applicable due to the small sample size investigated (“SR native”: Total dropouts [TDO] = 5/15 [33.3%] of which 60% were excluded due to weight loss [WL] and 40% were excluded due to sings of inflammation [SOI], images of one individual were excluded because of a dried out chamber; “SR nano”: TDO = 3/13 [23.1%], WL = 33%, SOI = 66%, images of two individual were excluded because of a dried out chamber and one due to insufficient image quality caused by non-adherence; “Etanercept native”: TDO = 3/12 [25.0%], WL = 33%, SOI = 66%; images of one individual were excluded because of a dried out chamber; “Etanercept nano”: TDO = 5/15 [33.4%], WL = 20%, SOI = 80%; images of one individual were excluded because of a dried out chamber, and one due to extensive hemorrhage within the chamber window).

### 3.2. Relative Fluorescence Intensity

The fluorescence intensity was investigated in all study cohorts, by fluorescence microscopy for a period of 10 days. In each animal, nine regions of interest within the observation windows of dorsal skinfold chambers of 8 C57BL/6J mice were examined daily. Off-line analysis allowed the exact quantification of fluorescence intensity which facilitated the calculation of the fluorescence kinetics over the investigated time period.

As described in detail in the section “Absolute Fluorescence Intensity” the fluorescence intensity immediately after PPE implantation significantly differed between study groups. As described in the Materials and Methods section in detail, to allow a statistical comparison of the kinetics regarding the decrease of fluorescence intensity in different investigational cohorts, the measured values were relativized to the baseline fluorescence after implantation to allow statistical comparability between individual cohorts and measurements as described in detail in the materials and methods section.

Previous experimentation revealed that native PPE as well as PPE modified with nanocarriers without any fluorescent cargo showed an fluorescence intensity within the range of background fluorescence (4–136 AU) in vivo (native PPE: 38–112 AU [*n* = 3]; PPE + nanocarriers [19–98 AU]).

**Figure 3:**
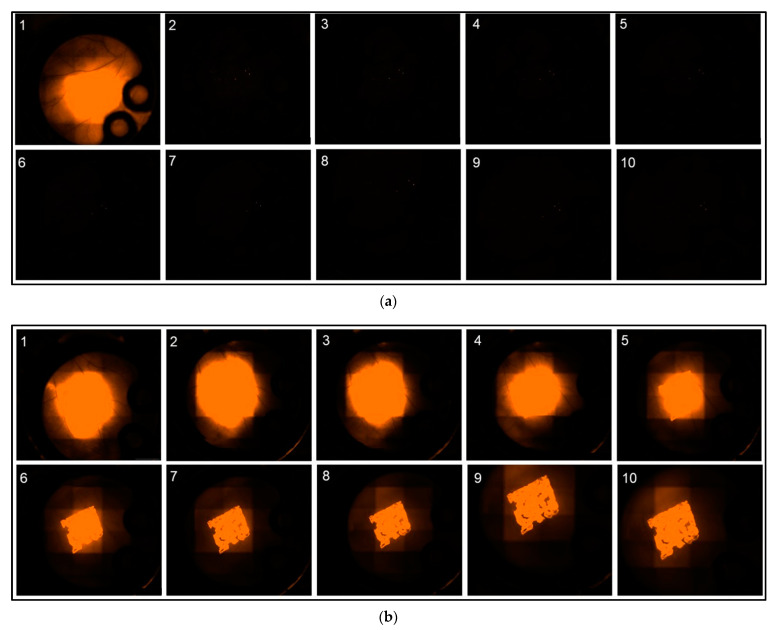
(**a**) Representative fluorescence microscopy images of “SR101 native” PPE over the investigated time period of 10 days, starting from day 1 (upper left corner) till day 10 (lower right corner) (White scale bars in the lower right corner of each image have a length of 2 mm). (**b**) Representative fluorescence microscopy images of “SR101 nano” PPE over the investigated time period of 10 days, starting from day 1 (upper left corner) till day 10 (lower right corner).

When analyzing the fluorescence intensity of the “SR native” cohort fluorescence intensity rapidly decreases post implantation (Figure 3a). In comparison, a significantly prolonged decrease was monitored in the “SR nano” cohort (Figure 3b).

**Figure 4:**
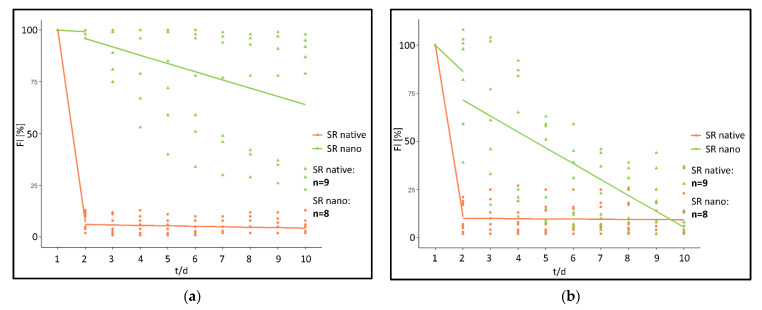
(**a**) Fluorescence course “SR native” and “SR nano” above the implant surface (ROI 1). Relative fluorescence intensity shown in percent (FI/%), time in days (t/d). (**b**) Fluorescence course “SR native” and “SR nano” in the chamber area directly adjacent to the PPE (ROI 2–5). Relative fluorescence intensity shown in percent (FI/%), time in days (t/d).

More specifically, in the “SR native” cohort within 24 h post implantation, fluorescence intensity had decreased by 92.78% at the implant surface (ROI 1) and 89.11% in ROI 2–5 directly adjacent to the PPE. After the initial reduction, fluorescence intensity further decreased by 0.22%/day (Implant surface) and 0.09%/day (chamber area directly adjacent to the PPE) during the period from day 2 post-implantation to day 10 (Figure 4).

In contrary, in the “SR nano” cohort, fluorescence intensity only decreased by 0.75% (ROI 1) and 13.63%/day (ROI 2–5) within the first 24 h post-implantation. Afterwards, the fluorescence intensity decreased by 3.98%/day (ROI 1) and 8.26%/day in ROI 2–5 during the investigated period from day 2–10 (Figure 4).

Using the linear mixed model, differences between the “SR native” and the “SR nano” cohort were calculated as follows.

For the first 24 h reduction of fluorescence intensity, determined by a difference of the linear gradient between the two investigated cohorts was statistically significant in ROI at the implant surface (*p* < 0.001) (difference: 92.03%/day). In ROI representing the chamber area directly adjacent to the PPE, a difference of 75.49%/day was calculated (*p* ≤ 0.001). For the investigated timeframe between day 2 and 5 significant differences between the investigated cohorts were calculated in ROI at the implant surface (*p* < 0.01) (difference: −3.76%/day). In ROI in the chamber area directly adjacent to the PPE a difference of 8.17%/day was calculated (*p* < 0.001).

Since values regarding the median fluorescence intensity in all study groups decreased to levels similar to the background fluorescence, further statistical analysis of ROI 6–9 (ROI distancing at least 200 µm from the implant surface) was omitted.

**Figure 5:**
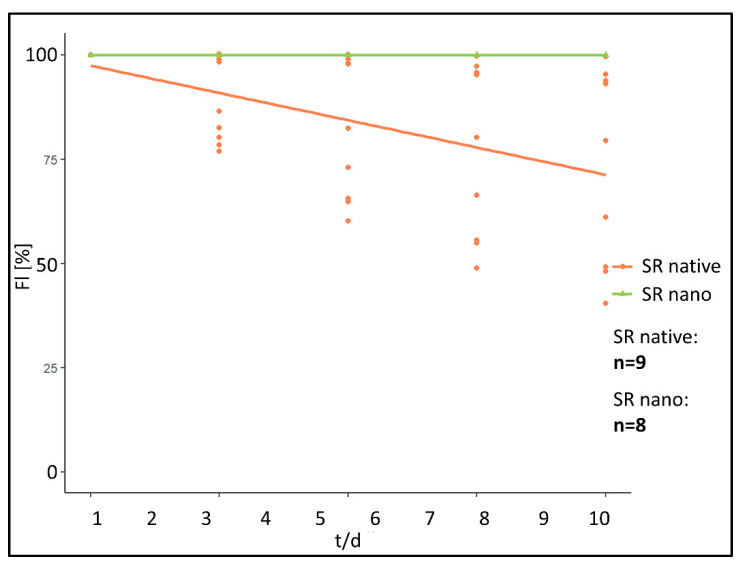
Fluorescence intensity kinetics “SR native” and “SR nano” in ROI at the implant surface, 50 ms, hour 1. Relative fluorescence intensity in percent (FI/%), time in minutes (t/min).

Since fluorescence intensity in the “SR native” cohort decreased rapidly within the first 24 h after implantation, additionally to the repetitive measures over 10 days the first hour after implantation was repetitively investigated every 15 min in the first 60 min after implantation (Figure 5).

Immediately after implantation, fluorescence intensity in the SR native cohort showed a decrease of relative fluorescence intensity of 0.44%/min in ROI at the implant surface, 0.46%/min in ROI in the chamber area directly adjacent to the PPE. In comparison, the “SR nano” cohort did not show any measurable decrease in ROI at the implant surface in the first 60 min after implantation (0.00%/min). In ROI in chamber area directly adjacent to the PPE, an increase in fluorescence intensity of 0.10%/min was measured.

Statistical comparison of fluorescence kinetics of both study groups during the first 60 min after implantation revealed dissimilarities in the kinetic for fluorescence intensity represented by a significantly different linear gradient for the calculated curves of the “SR native” and “SR nano” cohort (ROI at the implant surface [*p* < 0.01] [difference: 0.44%/min]. For ROI in the chamber area directly adjacent to the PPE, a difference of 0.56%/min was calculated [*p* < 0.01]).

**Figure 6:**
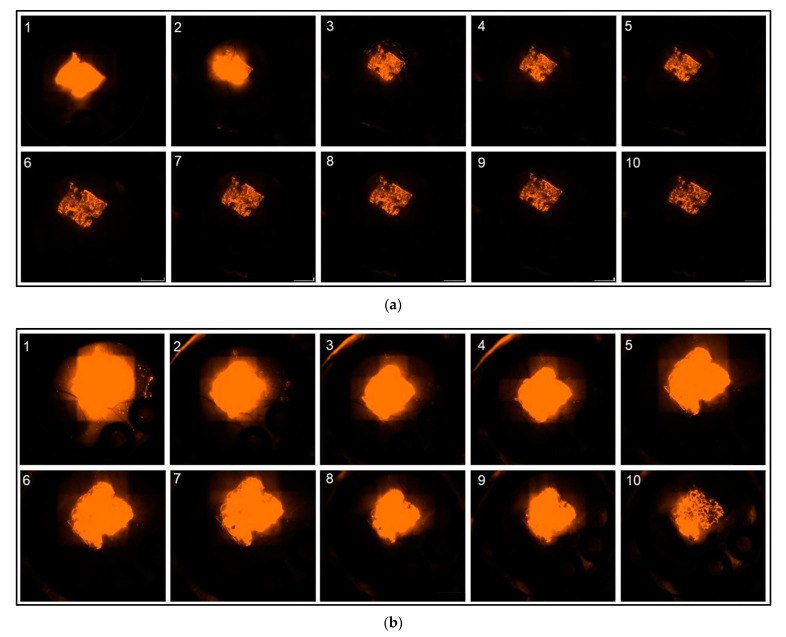
(**a**) Representative fluorescence microscopy images of “Etanercept native” PPE over the investigated time period of 10 days, starting from day 1 (upper left corner) till day 10 (lower right corner) (White scale bars in the lower right corner of each image have a length of 2 mm). (**b**) Representative fluorescence microscopy images of “Etanercept nano” PPE over the investigated time period of 10 days, starting from day 1 (upper left corner) till day 10 (lower right corner).

Analysis of the fluorescence intensity of modified implants, modified either with a non-encapsulated (“Etanercept native”) or encapsulated (“Etanercept nano”) macromolecule in ROI at the implant surface showed a visible fluorescence on day 10 of investigation in both investigated cohorts (Figure 6). Differences in fluorescence intensity and release kinetics did not differ significantly between both study groups.

**Figure 7:**
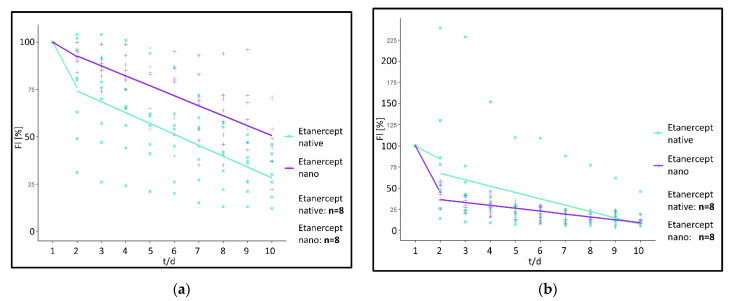
(**a**) Fluorescence course “Etanercept native” and “Etanercept nano” in ROI 1. Relative fluorescence intensity shown in percent (FI [%]), time in days (t/d). (**b**) Fluorescence course “Etanercept native” and “Etanercept nano” in ROI 2–5. Relative fluorescence intensity shown in percent (FI [%]), time in days (t/d).

More specifically, in the “Etanercept native” cohort, 24 h post implantation, fluorescence intensity decreased by 24.25% in (ROI 1) and 15.63% in ROI 2–5. After 24 h fluorescence intensity decreased by 5.75%/day (ROI 1) and 7.53%/day in ROI 2–5 in the period from day 2–10 (Figure 7).

In comparison, in the “Etanercept nano” cohort, 24 hrs post implantation, fluorescence intensity decreased by 7.75% (ROI 1) and 55.63%/day (ROI 2–5). Afterwards, the fluorescence intensity decreased by 5.27%/day (ROI 1) and 3.44%/day (ROI 2–5) in the investigated period from day 2–10 (Figure 7).

Using the linear mixed model, differences between the “Etanercept native” and the “Etanercept nano” cohort were calculated as follows.

For the first 24 h reduction of fluorescence intensity determined by a difference of the linear gradient between the two investigated cohorts was statistically not significant in ROI 1 (*p* = 0.09) (difference: 16.50%/day), and in ROI 2–5 a difference of −40.00%/day was calculated (*p* = 0.134). For the investigated timeframe between day 2 and 5 also no significant differences between the investigated cohorts were measurable in ROI 1 (*p* = 0.65) (difference: 0.48%/day). ROI 2–5 showed a difference of 4.09%/day (*p* = 0.15).

To characterize the fluorescence kinetics of native SR101 and native SR101-labeled Etanercept without the influence of the nanocarrier, both study groups were statistically compared using the linear mixed model. Comparison of the course of fluorescence intensity of native SR101 and native SR101-labeled Etanercept revealed differences regarding the kinetic of fluorescence intensity of the two substances in vivo (ROI 1: [*p* < 0.001] [difference: 68.53%/day]). For ROI 2–5 a difference of 73.48%/day was calculated (*p* < 0.01).

### 3.3. Values of Absolute Fluorescence Intensity

The intensity of the background fluorescence within the chamber window had median fluorescence intensity of 36 AU ranging from 4 to 136AU.

In the ROI at the implant surface, ROI 1 in all four experimental study groups, the fluorescence intensity on day 1 was significantly higher than the background fluorescence of the chamber and thus clearly distinguishable from the latter (data shown as median {AU} [range {AU}]) (“SR nano” = 4095 [4095 − 4095], “SR native” = 3594 [4095 − 507], “Etanercept nano” = 4081 [4095 − 4002], “Etanercept native” = 3918 [4083 − 3173] (Figure S2). The fluorescence intensity of ROI at the implant surface in all cohorts was clearly distinguishable from the background fluorescence also for the investigational period from day 2 to day 10. However, the “SR native” cohort showed fluorescence intensities similar to the background fluorescence after day 2 of investigation.

Fluorescence intensity in ROI in the chamber tissue adjacent to the PPE (ROI 2–5) on day 1 was comparatively lower than in the ROI at the implant surface, yet still exceeded the level of background fluorescence (“SR nano” = 3418 [4095 − 1908]; “SR native” = 976 [4094 − 83]. “Etanercept nano” = 1852 [3823 − 864]; “Etanercept native” = 1417 [3110 − 551]. Similar to the ROI at the implant surface, with the exception of the “SR native” cohort, during the investigated time period, the fluorescence intensity in ROI in the chamber tissue adjacent to the PPE still exceeded the intensity of background fluorescence.

As mentioned above, the fluorescence intensity in ROI 6–9 (Tissue distancing the PPE at least 200 µm) showed the lowest measured values on day one of investigation (“SR nano” = 412 [1570 − 62], “SR native” = 252 [2259 − 50], “Etanercept nano” = 276 [2262 − 66], “Etanercept native” = 414 [1612 − 78]. Since values regarding the median fluorescence intensity in all study groups decreased to levels similar to the background fluorescence further statistical analysis of ROI 6–9 was omitted.

In conclusion, prevalence of the small molecular SR101 on the implant surface, represented by a significantly lower decrease in fluorescence intensity over time, could be facilitated by encapsulation in albumin nanocarriers. Implants modified with native SR101 showed a significantly faster decrease in fluorescence within 24 h after implantation as well as in the first hour upon implantation compared to the implants modified with SR101 encapsulated in nanocarriers. Thus, bearing MSA nanocarriers showed a significantly prolonged fluorescence intensity over the investigated time period.

In comparison, implants modified with high-molecular SR101-labeled Etanercept showed a prolonged prevalence on the PPE and no significant differences between implants modified with native or encapsulated SR101-labeled Etanercept respectively were observed.

### 3.4. Analysis of the Vascular Network

MIA of the chamber window of *n* = 5 “SR native” mice as well as *n* = 6 mice of the “Etanercept native” and the “Etanercept nano” cohort each proved to be sufficient for further analysis. In all excluded images, either brightness of the implant fluorescence overexposed the chamber areas or the low contrast in the chamber window (“SR nano” cohort) impaired visualization in a way, that sufficient visualization of the vascular network was not possible.

**Figure 8:**
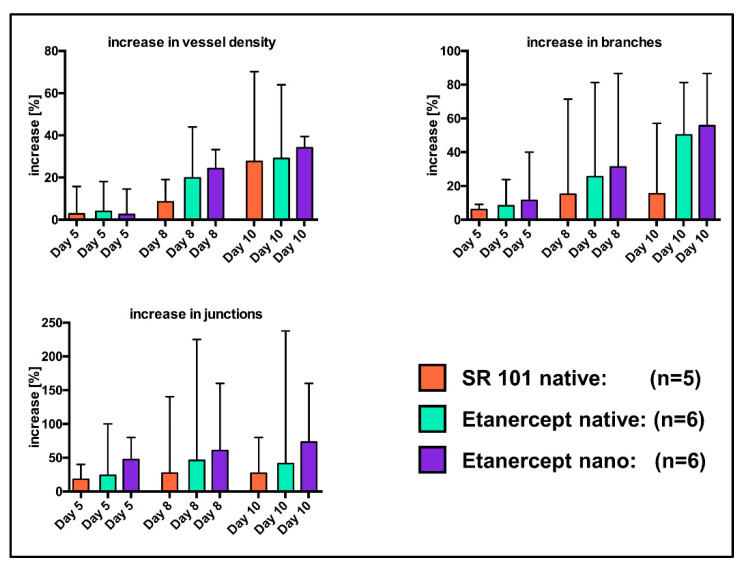
Relative increase in vessel density, vessel junctions and vessel branches. Data shown as median + range. Comparative analysis using the Mann Whitney *u* Test did not show any significant differences between study groups at the investigated time points.

Significant differences between cohorts were calculated neither for the percentual changes of vascular density, the number of vessel branches and vessel junctions (Figure 8), nor for the absolute values of these parameters (Figure S3), using the Mann Whitney *u* Test.

## 4. Discussion

Encapsulation in nanocarriers resulted in a significant alteration of the fluorescence kinetics of the small molecular dye SR101 whilst the fluorescence kinetics of the investigated, fluorophore labeled macromolecule did not show a difference reaching statistically significant levels during the investigated timeframe.

When looking at the values of absolute fluorescence intensity (Figure S2): Values were elevated compared to the background fluorescence in all experimental cohorts in the area of the implant surface and its adjacent surroundings. In the regions of the chamber further away from the PPE (ROI 6–9), the measured values were similar to the level of the background fluorescence. Since no changes in areas distancing the implant more the 100 µm were detectable, the assumption can be made that the coating of the PPE influences the area of the implant and its immediate surroundings, but not the more distant areas of the chamber window. This may indicate that implant modification with a pharmakon will result in high drug concentrations in an area distancing the implant surface 1000 µm or less but not tissues further away from the implant. Regarding values of absolute fluorescence, the variety in fluorescence intensity immediately after implantation upon individuals in a specific cohort is likely attributed to differences in implant structure, coating, implantation procedure of the PPE, and individual biological conditions within the chamber window. In this context, the measured intensity within the “SR native” cohort showed the widest range (Figure S2). Due to the fast clearance rate of SR101, very small differences regarding the investigational timepoint after implantation consequently lead to higher alterations in fluorescence compared to other study groups. Differences in fluorescence immediately after implantation between cohorts may further be caused by different concentrations of the dye in the nanocarriers and in the conjugate with Etanercept. An influence of autofluorescence, which has been previously described for the used nanocarriers (Em: 365 nm) [43,47], is unlikely due to the divergent spectrum used for intravital microscopy (Ex: 545 nm; Em: 605 nm). Furthermore, native PPE as well as PPE modified with nanocarriers without any fluorescent cargo was evaluated in vivo and showed a fluorescence intensity within the range of background fluorescence.

The analysis of fluorescence kinetics of implants coated with a molecule with low molar mass (“SR native” cohort) showed an intensity within the range of the background fluorescence of the chamber, both in the ROI at the implant surface as well as the ROI directly adjacent to the PPE 24 h after implantation, indicating the absence of the fluorescent dye in measurable concentrations.

The rapid decrease in fluorescence intensity in the “SR native” cohort can likely be explained by rapid diffusion and degradation of the fluorescent dye. For many small and unstable growth factors similar kinetics can be assumed [28,32,33,53]. The fast clearance of native SR101 is further supported by the significant decrease in fluorescence intensity in the first hour after implantation, compared to the encapsulated SR101 (Figure 5). Small molecular substances exhibit a particularly high diffusion rate due to small size [54]. Contrary, the release of a substance from micro- or nanocarriers is generally determined by the desorption of substance bound or adsorbed on the surface, the diffusion of the substance through the particle matrix, the degradation of the matrix, and the combination of diffusion and matrix degradation. This theory is supported by the finding that the short half-life of a low molar substance like SR101 in the microenvironment of the implant can be prolonged by encapsulation in MSA nanocarriers and electrostatical binding of the latter on the implant surface (Figure 3, Figure 4 and Figure 5).

Additionally, for the investigation of encapsulation of a low molar substance, effects on the encapsulation of a high molecular substance were investigated. SR101-labeled Etanercept was chosen as a representative substance due to its large molecular size (~150 kDa) and promising effects regarding the inflammatory response of PPE implants in vivo as recently described [55]. Intravital microscopy showed optically visible fluorescence of the PPE implants in both groups throughout the entire experimental period (Figure 6). Consequently, a presence of Etanercept in measurable concentration on the implant surface after ten days is present regardless of the specific experimental cohort. Hence, no difference reaching statistical significance was detected between the experimental cohorts above the implant surface (ROI 1) and in the implant environment (ROI 2–5) during the investigation period.

Comparison of the course of fluorescence intensity of the low molecular native SR101 and the native high molecular SR101-labeled Etanercept showed a different kinetic of fluorescence intensity. SR101 (606.71 Da) and Etanercept (~150 kDa) differ significantly in molecular weight and size, which critically affects diffusion behavior. Accordingly, an altered diffusion rate of Etanercept from the implant surface may at least partially be attributed to an alternate diffusion rate compared to the small SR101. Dependence of the release of substances on their molecular weight has also been established in various other experiments [34,56,57]. In addition to the slower clearance due to the high molecular weight, Etanercept also has an in vivo half-life of approximately 70 h (Pfizer Pharma GmbH, 2019) far exceeding the half-life of SR101 which has a reported in vivo half-life of 1–2 h after intravenous injection [58]. Albumin on the other hand has a reported half-life of approximately 19 days [39]. Thus, the influence of albumin nanocarriers on the pharmacokinetics of the stable Etanercept was estimated to be significantly lower than the effect of albumin on the pharmacokinetics of the smaller and more unstable SR101.

Regarding the release and degradation, potential drug-associated effects have also to be taken into consideration. SR101-linked Etanercept is a potent drug, which may affect the inflammatory tissue response to the implants and thus could also influence the release kinetics by alternating the recruitment of cells and vessels in the implant. In that regard, application of a non-functional macromolecule would have allowed the exclusion of potential drug-associated effects, however, preliminary experiments evaluating green fluorescent protein as cargo vs. nanocarriers without any encapsulated fluorophore revealed similar fluorescence kinetics compared to the fluorescence kinetics of encapsulated Etanercept (Figure S4). However, since degradation of an alternate fluorophore differentiates from the degradation of SR101, using an alternate fluorophore ultimately results in a similar conceptual weak point regarding the comparability of fluorescence kinetics.

Regarding the influence of drug-associated Etanercept on fluorescence intensity, looking at the specific effects on vessel density might be of value. Implant modification with encapsulated or non-encapsulated Etanercept resulted in no statistically significant differences regarding the vascular density or the number of vessel junctions or branches (Figure 8 and Figure S3). We attribute the absence of any biological effects with the hypothesis that fluorescence labeling with SR101 might interfere with the biological function of Etanercept thus impairing effects on the vascular network. Furthermore, considering the stability of the albumin nanocarriers, evaluation of specific effects of encapsulation might also be influenced by the limited observation period when using the dorsal skinfold chamber model. Additionally, the observational quality can be improved by intravascular application of fluorophores allowing a more precise evaluation of the vascular network [23,48,49,55]. Therefore, further experiments evaluating the influence of surface modification with albumin nanocarriers on biological effects are urgently needed and longer observation periods should be considered to determine the specific effect of encapsulation especially when high molar substances are encapsulated.

Evaluation of fluorescence kinetics of encapsulated and not-encapsulated Etanercept did not show statistically significant differences between the cohorts. Although a tendency of a prolonged release can be observed (Figure 7), the differences did not reach statistical significance (*p* = 0.09 [ROI 1]; *p* = 0.134 [ROI 2–5]). Considering the long half-life of both the encapsulated substance as well as the albumin nanocarriers, based on the data obtained in our experiments, a definite and well-founded statement regarding the influence of the albumin nanocarriers on the release kinetics of Etanercept cannot be made and is ultimately impaired by the variation in fluorescence intensity and the limited observation period, when using the dorsal skinfold chamber model [59,60]. Models that allow a longer observational timeframe might be more suited to properly investigate differences in release kinetics in this specific case. Subcutaneous implantation would allow a significantly longer observation period, yet establishing imaging techniques such as in vivo bioluminescence imaging would be a prerequisite to visualize the factor release in vivo.

The utilization of albumin nanocarriers as DDS has previously been investigated by other working groups, however, most investigated albumin particles showed a rather fast degradation in vitro and in vivo [42,61]. Depending on the specific albumin particles used, albumin nanocarriers were able to achieve a delayed, biphasic release of the encapsulated pharmaceuticals within hours. As vascularization of implants take place within a period of several days to weeks [62,63,64,65,66] and effects of pro-angiogenic and growth modulating factors are often limited by short half-lives [32,67,68,69], an ideal DDS should be able to maintain the concentration of encapsulated substances for a much longer timeframe, analogous to the duration of vascularization and tissue ingrowth. Consequently, the release kinetics of albumin nanocarriers described in most other publications are not suited to maintain a sustained release for a sufficiently long time period in the field of tissue engineering.

Taking these prerequisites into consideration, preliminary in vitro studies of the albumin nanocarriers, crosslinked by orthogonal photoclick tetrazole–ene chemistry used in this study demonstrated no leakage of SR101 from the nanocarriers for 78 days [47]. The albumin shell completely prevented diffusion of the encapsulated dye and remained stable for the investigated timeframe. Under the influence of trypsin, the capsule was enzymatically degraded and SR101 was released from the nanocarriers [47,70]. With the in vivo experiments we were able to elucidate corresponding results under physiological conditions in vivo. The albumin nanocarriers limited the release of small molecular fluorophores within the skinfold chamber by maintaining the presence of the fluorescent dye in the tissue for at least ten days, which indicates, that degradation of the particles in vivo takes several days to weeks. Despite the limited investigation period of only 10 days, the results obtained with crosslinked nanocarriers in vivo show a prolonged presence of small-molecule active substances in the implant and adjacent tissues. Thus, modification of the implant surface with these albumin nanocarriers should allow the mediation of long-term proangiogenic and/or immunomodulating effects.

In the field of tissue engineering, apart from albumin, gelatin [71], chitosan, or alginate [72,73], the most widely researched biodegradable and biocompatible polymers are the poly(α-ester)s like poly(lactic acid) (PLA), poly(glycolic acid)(PGA), and copolymers of these which have shown their potential to realize a prolonged drug release and, in some cases, promising effects in terms of enhancing tissue regeneration, ingrowth, and promotion of angiogenesis [72,74,75,76]. However, their synthetic origin implies several drawbacks like fast clearance by the immune system, inflammatory reactions [77,78], or even toxicity [79,80]. Furthermore, negative effects on the efficacy of the released pharmaceuticals have also been described and can probably be attributed to the acidic micromilieu in the particle environment caused by degradation [81,82]. Negative effects associated with particle degradation may result not only in the instability of released proteins [83] but can also trigger an inflammatory response in tissues and subsequently cause impairment of wound healing due to the acidic degradation products of the polymers [31,84,85]. Taking these findings into consideration, surface modification with albumin nanocarriers has obvious advantages [47] if sufficiently long time period of release can be realized. To avoid rapid degradation, Zhang et al. used coating of albumin nanospheres with polyethyleneimine to stabilize them which resulted in a significantly slower release compared to uncoated nanospheres in vitro and in vivo [86]. The polyethyleneimine-coated albumin nanoparticles were used to modulate the pharmacokinetics of bone morphogenetic protein 2 (BMP-2). However, an effect of BMP-2 in the form of bone growth could not be observed which was attributed to the toxicity of polyethylenimine [86]. Contrary to these findings, for the specific nanocarriers used in our experiments, no evidence of toxicity was demonstrated in preliminary experiments [47].

Beyond the advantages in regard to immunogenicity, toxicity, and influences on the encapsulated substances, particles could be sufficiently bound to PPE with electrostatical interactions without the need for further modification. As PPE is an inert substance other implants from materials forged from titanium, silicones, polyethylene terephthalate (Dacron^®^) or PLGA and PLA can likely be realized and broaden the applicability of the albumin nanocarriers for various applications.

## 5. Conclusions

Complementing the promising results in vitro, our data highlight crosslinked albumin nanocarriers as a capable tool for the modification of the implant surface in the field of tissue engineering, especially when small molecular substances are to be administered.

## Figures and Tables

**Table 1:** Amounts of reagents for modification of MSA with TET.

Reagent	Molar Mass/g/mol	Mass	Molar Amount/mol	Equivalents
Mouse serum albumin	68,692.5	50 mg	7.28 × 10^−7^	1.0
TET	266.26	0.99 mg	3.71 × 10^−6^	5.1
DMAP	122.17	0.045 mg	3.71 × 10^−7^	0.51
EDC	191.7	0.71 mg	3.71 × 10^−6^	5.1
DMSO	78.13	3 mL	solvent	solvent

## Data Availability

Detailed data regarding the results of in vivo experimentation as well as the methodological procedure is described in detail in Niklas Hoorman’s doctoral thesis entitled “Albumin-Nanopartikel als Drug Delivery System zur Oberflächenmodifikation poröser Polyethylenimplantate”. The Doctoral thesis in written form is available in the Universitätsbibliothek Mainz.

## Supplementary Materials

The following are available online at https://www.mdpi.com/article/10.3390/biomedicines9101485/s1, Figure S1: Representative fluorescence microscopy images of evaluation of the the vascular network after implantation. Figure S2: Median values of fluorescence intensity (Fluor [in Arbitrary Units {AU}]) are shown for each investigated cohort in the Regions of interest (ROI) at the implant surface as well ROI in the chamber tissue adjacent to the PPE and ROI distancing the implant at least 200 µm. Figure S3: Absolute values of vessel density, vessel junctions and vessel branches. Figure S4: Course of fluorescence intensity in dorsal skinfold chambers of 4 mice after implantation of PPE modified with nanocarriers encapsulating Green fluorescent Protein (GFP) or nanocarriers without any encapsulated substance.
