# Supplementary material for: Surface Modification of Porous Polyethylene Implants with an Albumin-Based Nanocarrier-Release System
Source: Biomedicines. 2021 Oct 16;9(10):1485. doi: 10.3390/biomedicines9101485 (PMC8533240; doi:10.3390/biomedicines9101485)
Supplement: Supplementary file 1 [file biomedicines-09-01485-s001.zip › biomedicines-1383757-supplementary.pdf]

biomedicines

MDPI

Surface Modification of Porous Polyethylene Implants with an Albumin-Based Nanocarrier-Release System

Supplementary Materials

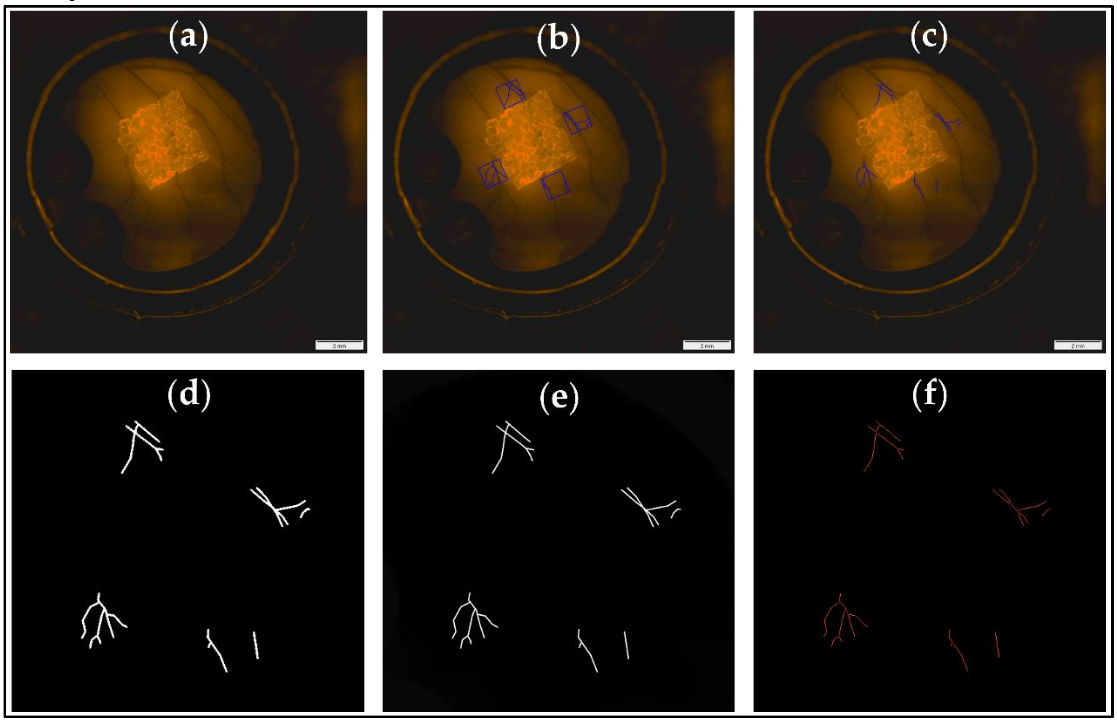

Figure S1. Representative fluorescence microscopy images of evaluation of the the vascular network after implantation. MIA were reduced in brightness and overall contrast using cellSens Dimension (OLYMPUS DEUTSCHLAND GmbH, Hamburg, Germany) till detection of the vascular network was possible. Images of day 1, 5, 8 and 10 after implantation where then reevaluated regarding vascular density, if the vascular network was visible and image proved to be sufficient for further analysis (**a**). The vascular network within the ROI directly adjacent to the implant (ROI 2–5) was then traced by hand using the “Polyline” function in cellSens Dimension thus measuring the vascular density (vessel length/mm^2) in every ROI (**b**). Images where subsequently exported as jpg. after removal of the ROI frame (**c**). After cropping of the image, the blue channel was isolated using IrfanView (**d**). The images were imported into the Java-based open-source software Fiji. Image were converted into black and white using Fiji`s “Make binary” function and subsequently Skeletonized using the “Skeletonize” function (**e**). The Skeleton was then Analyzed using the “Anlalyze Skeleton” function and values of branches and junctions (**f**) of every image ware calculated and exported into Excel files.

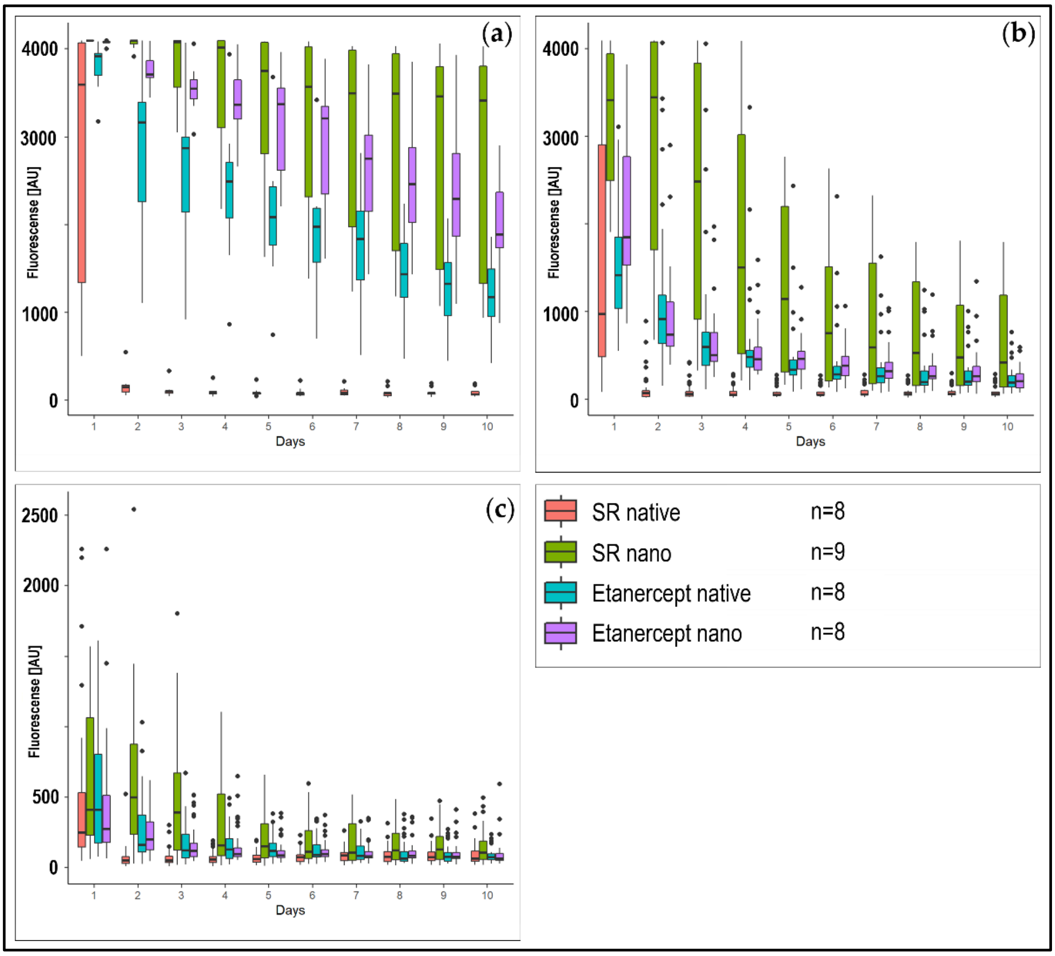

Figure S2. Median values of fluorescence intensity (Fluor [in Arbitrary Units {AU}]) are shown for each investigated cohort in the Regions of interest (ROI) at the implant surface (**a**) as well ROI in the chamber tissue adjacent to the PPE (**b**) and ROI distancing the implant at least 200 µm (**c**). Data shown as Median +/− Range. The range of detectable fluorescence intensity ranged from 0 AU to a maximum of 4096 AU.

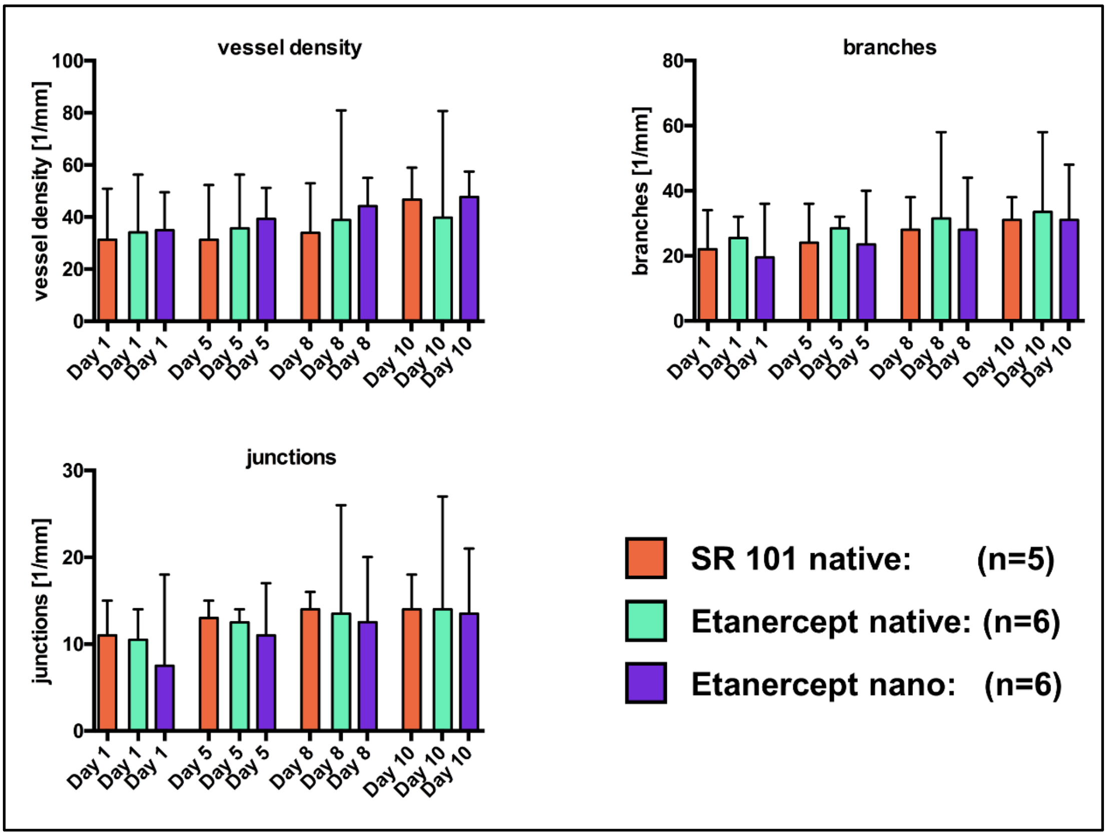

Figure S3. Absolute values of vessel density (**a**), vessel junctions (**b**) and vessel branches (**c**). Data shown as Median + Range. Comparative analysis using the Mann Whitney *u* Test did not show any significant differences between study groups at the investigated time points.

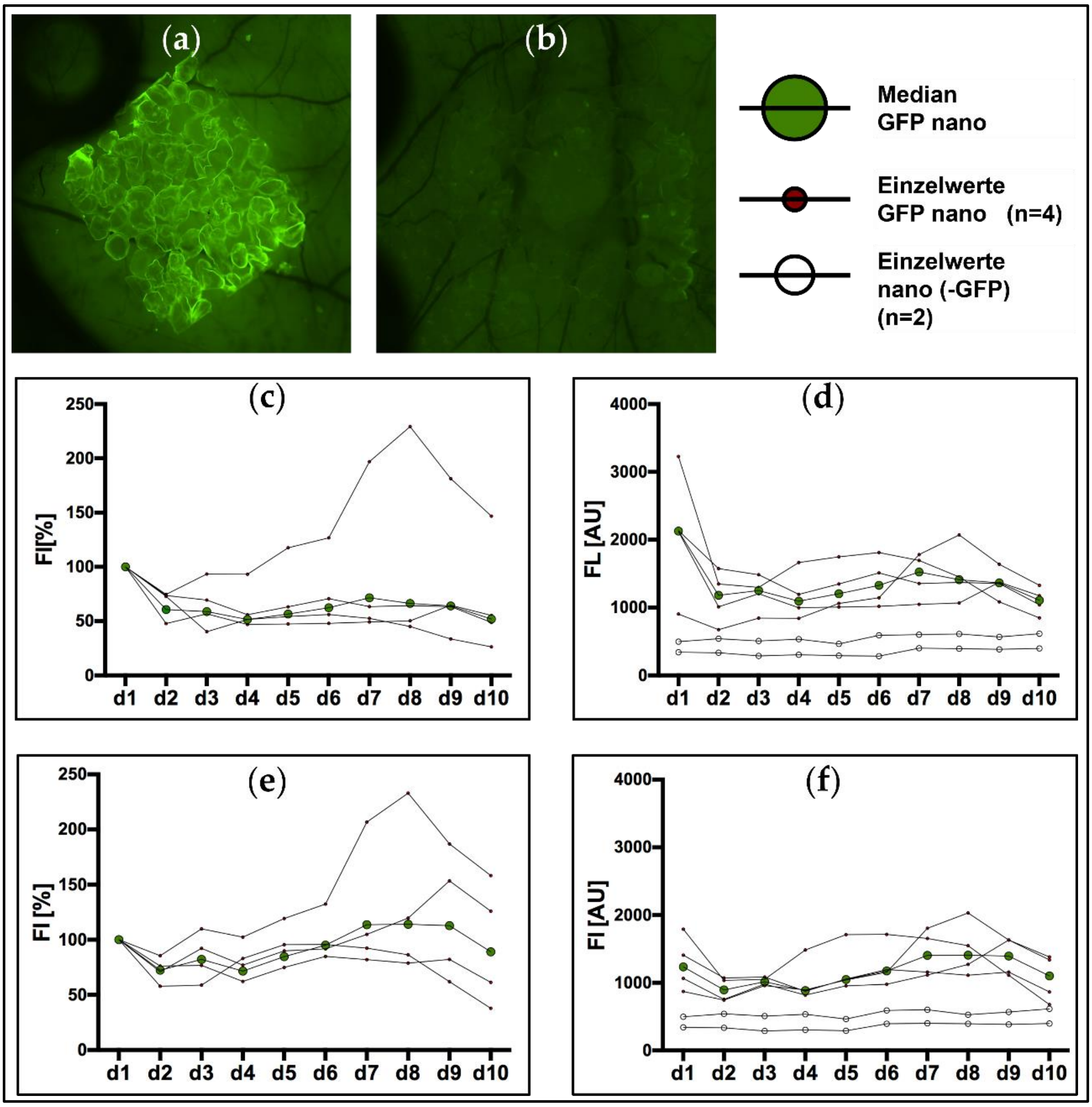

Figure S4. Course of fluorescence intensity in dorsal skinfold chambers of 4 mice after implantation of PPE modified with nanocarriers encapsulating Green fluorescent Protein (GFP) (**a**) or nanocarriers without any encapsulated substance (**b**). Relative fluorescence intensity (%) as well as absolute Fluorescence intensity in arbitrary units (AU) is shown. Relative fluorescence intensity over the implant surface (**c**) as well as the surrounding tissue (**e**) as well as absolute fluorescence intensity over the implant surface (**d**) as well as the surrounding tissue (**f**) is.
